# Enhancement of X-ray detection by single-walled carbon nanotube enriched flexible polymer composite

**DOI:** 10.1186/1556-276X-9-610

**Published:** 2014-11-12

**Authors:** Heetak Han, Sanggeun Lee, Jungmok Seo, Chandreswar Mahata, Sung Hwan Cho, A-Reum Han, Keun-Sung Hong, Joon-Ho Park, Myung-Jin Soh, Cheolmin Park, Taeyoon Lee

**Affiliations:** Nanobio Device Laboratory, School of Electrical and Electronic Engineering, Yonsei University, 50 Yonsei-ro, Seodaemun-Gu, Seoul, 120-749 Republic of Korea; Nano-Polymers Laboratory, Department of Materials Science and Engineering, Yonsei University, 50 Yonsei-ro, Seodaemun-Gu, Seoul, 120-749 Republic of Korea; Luxen Technologies, Inc., 396 Worldcupbuk-ro, Mapo-Gu, Seoul, 121-795 Republic of Korea

**Keywords:** Semiconducting polymers, X-ray detectors, Single-walled carbon nanotubes, Flexible electronics

## Abstract

**Abstract:**

Although organic-based direct conversion X-ray detectors have been developed, their photocurrent generation efficiency has been limited by recombination of excitons due to the intrinsically poor electrical properties of organic materials. In this report, we fabricated a polymer-based flexible X-ray detector and enhanced the X-ray detection sensitivity using a single-walled carbon nanotube (SWNT) enriched polymer composite. When this SWNT enriched polymer composite was used as the active layer of an X-ray detector, it efficiently separated charges at the interface between the SWNTs and polymer, preventing recombination of X-ray-induced excitons. This increased the photocurrent generation efficiency, as measured from current-voltage characteristics. Therefore, X-ray-induced photocurrent and X-ray detection sensitivity were enhanced as the concentration of SWNTs in the composite was increased. However, this benefit was counterbalanced by the slow and unstable time-dependent response at high SWNT concentrations, arising from reduced Schottky barrier heights between the active layer and electrodes. At high SWNT concentration, the dark current also increased due to the reduced Schottky barrier height, leading to decrease the signal-to-noise ratio (SNR) of the device. Experimental results indicated that 0.005 wt.% SWNT in the composite was the optimum composition for practical X-ray detector operation because it showed enhanced performance in both sensitivity and SNR. In mechanical flexibility tests, the device exhibited a stable response up to a bending radius of 0.5 cm, and the device had no noticeable change in diode current after 1,000 bending cycles.

**PACS code:**

8.67.Sc

**Electronic supplementary material:**

The online version of this article (doi:10.1186/1556-276X-9-610) contains supplementary material, which is available to authorized users.

## Background

Since their development, radiation detectors have been widely used in various fields such as crystallography, medical imaging, and security due to their ability to inspect visually opaque objects [1–3]. Conventionally, the active layers of X-ray detectors have been composed of inorganic materials such as cadmium telluride (CdTe), silicon carbide (SiC), and amorphous selenium (a-Se), since they offer high energy resolution, high detection efficiency, and room-temperature operation [4, 5]. However, inorganic-based X-ray detectors also suffer from some critical drawbacks in terms of large-scale fabrication, high cost, and fragile property. Especially, it is difficult to make flexible devices based on inorganic materials, and although flexibility can be achieved by using a thin inorganic layer, this reduced the active layer thickness and thus sacrifices absorption ability [6].

In recent decades, organic-based semiconducting materials have been widely used as active layers of various electronic devices such as photovoltaic devices [7], light-emitting diodes [8], and thin-film transistors [9] due to their relatively low cost, availability for large-area fabrication, and mechanical flexibility [10]. However, there have been only a few scientific publications on the use of semiconducting organic materials for direct detection of X-rays. Boroumand et al. reported the first direct X-ray detection of X-ray-induced photocurrents in thick films of conjugated polymers using poly[1-methoxy-4-(2-ethylhexyloxy)-phenylenevinylene] (MEH-PPV) and poly(9,9-dioctylfluorenyl-2,7-diyl) (PFO) [11]. Intaniwet et al. reported a direct X-ray detector using blends of polymer poly(triarylamine) (PTAA) and 6,13-bis(triisopropylsilylethynyl) (TIPS)-pentacene to increase the transport of holes [12]. Despite these efforts, however, the X-ray-induced output photocurrents of such devices are still limited due to the intrinsically low electron carrier mobility of the organic materials [13].

Herein, we demonstrated a polymer-based flexible X-ray detector and enhanced the X-ray detection sensitivity using single-walled carbon nanotubes (SWNTs). Because a SWNT has extremely high electron mobility and greater electron affinity than a p-type semiconducting polymer, the X-ray-induced excitons generated in the SWNT enriched polymer composite can be effectively separated and guided toward their respective electrodes without recombination. The composite was coated onto a poly(ethylene terephthalate) (PET) substrate to fabricate the active layer of the device, and homogenous dispersion of the SWNTs was confirmed through optical microscope and scanning electron microscope (SEM) images. Current-voltage (*I*-*V)* characteristics and X-ray-induced photocurrents of the devices were measured, and we verified that increasing the concentration of SWNTs in the composite layer not only enhanced the X-ray-induced photocurrent and X-ray detection sensitivity but also reduced the response speed and stability of the device. The optimum SWNT concentration was determined in consideration of both sensitivity and signal-to-noise ratio (SNR) of the device. Since SWNTs have exceptional mechanical properties and polymers are composed of cross-linked molecules, mechanical flexibility of the X-ray detector was achieved without noticeable degradation.

## Methods

### Materials

Metallic SWNTs (Hanwha Nanotech, Daejeon, Korea) with 1 to 1.2 nm in diameter, 5 to 20 μm in length, and 70 wt.% purity were used in this work. Poly(styrene-*b*-paraphenylene) with polyphenylene rich in 1,4-addition (PS-*b*-PPP) was synthesized via dehydrogenation of poly(styrene-*b*-1,4-cyclohexadiene) (Polymer Source, Dorval, Canada). The p-type semiconducting polymer, ‘Super Yellow’ (SY, Merck, Darmstadt, Germany), was used without purification.

### Preparations of solutions of SWNT enriched polymer composite

The detailed sample preparation process of composite solutions is described elsewhere [14]. In brief, we prepared 1 mg/mL of PS-*b*-PPP solution in toluene. Then, 1 mg/mL of SWNT solution in PS-*b*-PPP/toluene was prepared by adding SWNTs into PS-*b*-PPP/toluene. The mixture was horn-sonicated for 5 min (VC 750, Sonics & Materials, Newtown, CT, USA), followed by a 10-min bath sonication (NXP-1002, Kodo Technical Research, Hwaseong, Korea). SWNT solutions with various concentrations were made by diluting the 1 mg/mL SWNT solution in PS-*b*-PPP/toluene with pure toluene. SYs (10 mg/mL) were directly dissolved into diluted SWNT solutions to prepare the SWNT/SY composite solution. The resulting SWNT concentrations ranged from 0 to 0.1 wt.% for the SY polymer. The whole process was carried out under room temperature.

### Fabrications of SWNT enriched polymer composite-based flexible X-ray detector

A 5-μm-thick SWNT enriched polymer composite film was prepared by successive drop casting onto PET substrates with predefined Au electrodes (60 nm). The coated films were stored at room temperature for an hour until toluene fully evaporated. Finally, LiF (5 nm) and Al (60 nm) were sequentially deposited on the composite layer by thermal evaporation. The defined active area of the devices was 3 × 3 mm^2^.

### Characterizations

*I*-*V* characteristics in the dark and under X-ray illumination were measured using a Keithley 2400 SourceMeter (Keithley Instruments Inc., Cleveland, OH, USA). X-ray photocurrent measurements were carried out with an 8.06-keV Kα X-ray generated from a copper target X-ray tube. The X-ray beam was irradiated directly onto and through the Al top electrode at room temperature. The morphology of the composite layer was characterized by a field-emission scanning electron microscope (JSM-7001f, JEOL, Tokyo, Japan).

## Results and discussion

**Figure 1:**
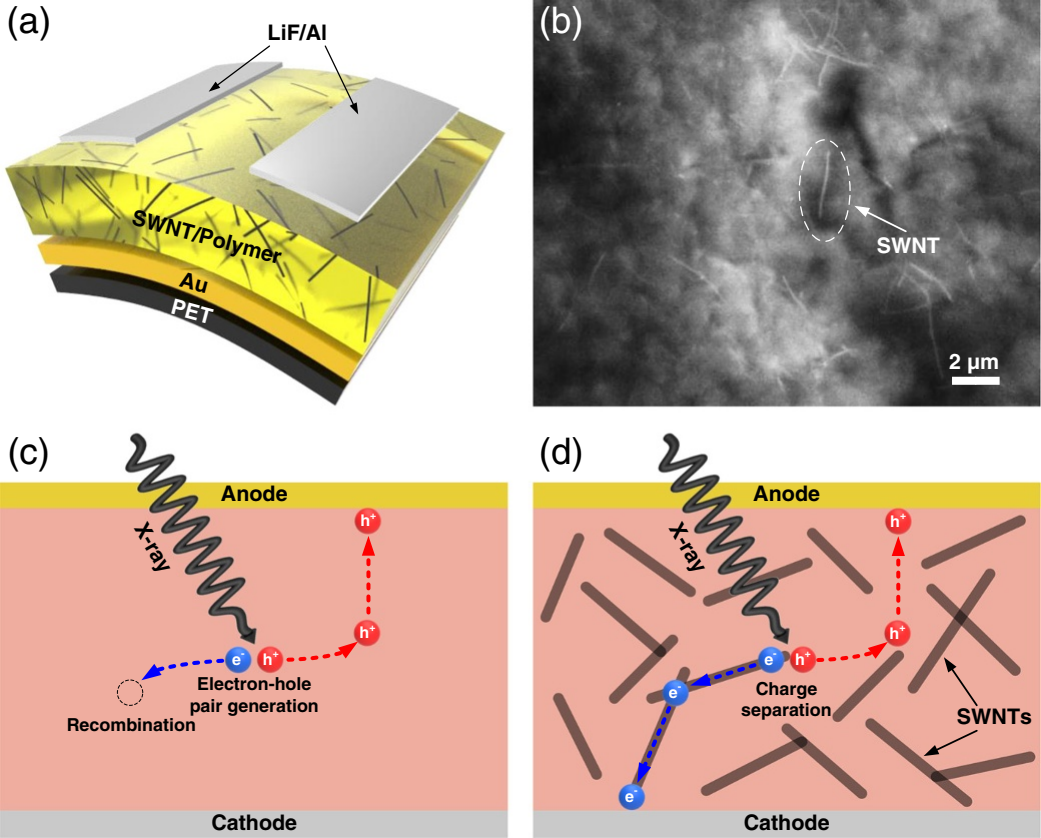
**Schematic illustrations and SEM image of the fabricated devices. (a)** Schematic illustration of the fabricated flexible X-ray detector structure and **(b)** SEM image of the composite layer (0.1 wt.% SWNT concentration). Schematic illustration of charge separation in **(c)** a pure p-type polymer device and **(d)** a SWNT enriched polymer composite device.

Figure 1a schematically illustrates the SWNT enriched polymer composite-based flexible X-ray detector. In this report, we used PS-*b*-PPP, a conjugated block copolymer, as a dispersant, which allows a uniform distribution of SWNTs in both the solution and composite film [14]. The distribution of SWNTs in the composite film was observed by using the SEM image. Figure 1b shows a top-view SEM image of the SWNT enriched polymer composite film at 0.1 wt.% SWNT concentration, and there was no observation of severe agglomeration of SWNTs in the composite. In addition, we also confirmed the distribution of SWNTs in both the solution and composite film through optical images (Figure S1 and S2 in Additional file 1). Without the dispersant, SWNTs in the toluene solution were bundled to each other and sank to the bottom of the vial within a few minutes. However, with the dispersant, SWNTs were well-dispersed in toluene solution and no aggregation was observed even after 1 month of storage at room temperature (Figure S1 in Additional file 1). As shown in Figure S2 in Additional file 1, the composite film without the dispersant showed a large aggregation of SWNTs, whereas the composite film with the dispersant showed a smooth film morphology without any dark spots.

Figure 1c,d illustrates the charge separation mechanism in the devices. It has been reported that when a pure p-type conjugated polymer is used as the active layer for an X-ray detector, it produces low photocurrent because of its low electron mobility and high recombination rate; this mechanism is illustrated in Figure 1c [15]. As illustrated in Figure 1d, after SWNTs are added to the active layer, charge separation can easily occur at the SWNT-polymer interface. Electrons move into the SWNTs and holes move into the polymer because the SWNTs have a greater electron affinity than the polymer [16]. This effective charge separation prevents the recombination of charges, enhancing the X-ray-induced photocurrent [17].

**Figure 2:**
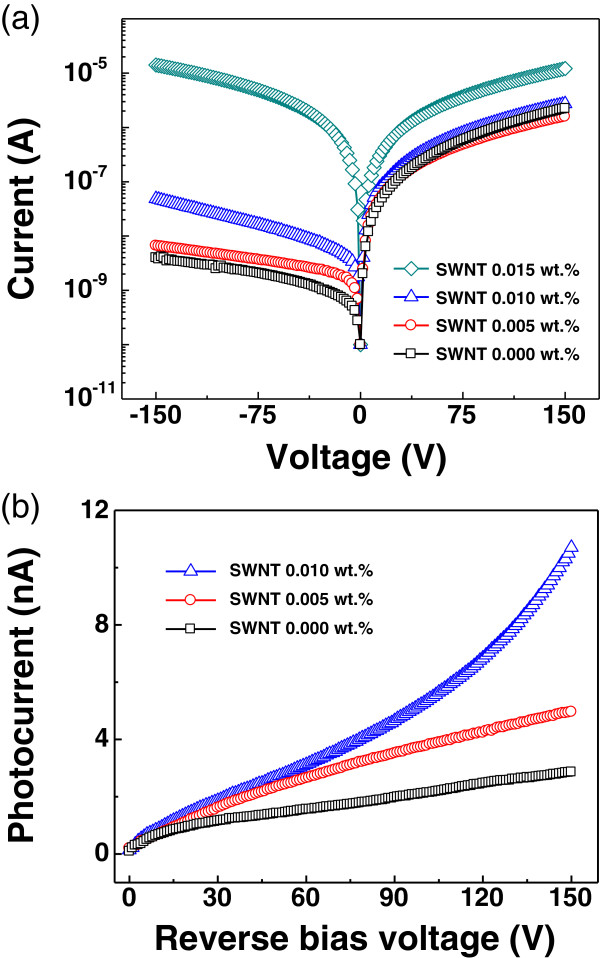
**Current-voltage characteristics and photocurrents of the fabricated devices. (a)** Dark current-voltage characteristics of flexible X-ray detectors with various SWNT concentrations. Ambipolar characteristics were observed as SWNT concentration was increased. **(b)** Photocurrents as a function of applied reverse bias voltage for devices with different SWNT concentrations. Photocurrent was enhanced with increasing SWNT concentration up to 10.67 nA at 150 V for the 0.010 wt.% SWNT device.

Figure 2a shows the dark *I*-*V* characteristics of the devices with various SWNT concentrations under voltages ranging from -150 to 150 V applied to the Au electrode. In the case of the polymer device without SWNTs (0.000 wt.% SWNT), the *I*-*V* curve showed rectifying behavior with low reverse bias current. As the SWNT concentration was increased from 0 to 0.01 wt.%, the resulting *I*-*V* curves of the devices still showed similar rectifying behavior. However, after the concentration of SWNTs was increased above 0.015 wt.%, the device showed ambipolar characteristics. At excessively high carbon nanotube concentrations, it is known that electrical properties of devices are determined by the carbon nanotubes than the polymer [18]. Under the domination of SWNTs, it is natural that the electrical property of the device shows ambipolar characteristic because the SWNT we used has metallic property. To verify this, we also fabricated a similar device using the composite of insulating poly(methyl methacrylate) (PMMA) and SWNT as the active layer. As shown in Figure S3 in Additional file 1, the *I*-*V* characteristic of the SWNT/PMMA device showed ambipolar characteristics at a high SWNT concentration (0.1 wt.%).

**Figure 3:**
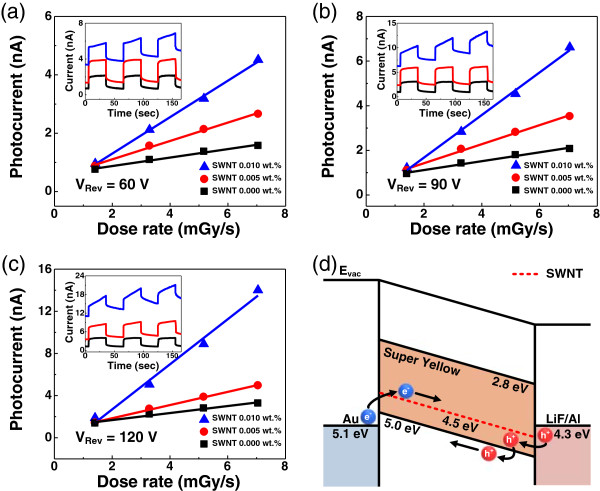
**Photocurrents and band diagram of the fabricated devices.** Photocurrents of devices with three different SWNT concentrations as a function of applied X-ray dose rate under the reverse bias voltages of **(a)** 60 V, **(b)** 90 V, and **(c)** 120 V. Insets show the devices' time-dependent responses. **(d)** Band diagram of the flexible X-ray detector. Charges can be easily injected into the active layer through the reduced Schottky barrier between the active layer and electrodes.

Figure 2b shows the X-ray-induced photocurrent as a function of reverse bias voltage for the devices with three different SWNT concentrations. The reverse bias voltage applied ranged from 0 to 150 V with an X-ray dose rate of 7 mGy/s. The photocurrents were calculated by subtracting the dark current from the X-ray irradiated current. It was clearly observed that the photocurrents of the X-ray detectors were increased for all applied operational voltages when the SWNTs were included in the active layer. Especially, under high electric field, the photocurrents were considerably increased as the SWNT concentration increases. For instance, at a reverse bias voltage of 150 V, the photocurrents of the devices increased from 2.86 to 10.67 nA, which is about 273% larger, by increasing the SWNT concentration from 0 to 0.01 wt.% as listed in Table 1. When the reverse bias voltage increased, the devices also showed different photocurrent increase tendencies depending on the concentration of SWNT. The 0.000 wt.% SWNT and 0.005 wt.% SWNT devices showed a saturating photocurrent tendency, whereas the 0.010 wt.% SWNT device showed a non-saturating photocurrent tendency. These differences according to the SWNT concentration were possibly due to charge injection from the electrodes caused by a reduction in the Schottky barrier height between the active layer and each electrode at a high SWNT concentration (this will be discussed in more detail in the next section; see also Figure 3d).

**Table 1:** **Photocurrents and enhancements of the devices**

SWNT concentration (wt.%)	Photocurrent (nA)	Enhancement (%)
0.000	2.86	-
0.005	4.96	Approximately 73
0.010	10.67	Approximately 273

Photocurrents and enhancements of the devices with three different SWNT concentrations at a reverse bias voltage of 150 V.

Figure 3a,b,c illustrates the relationship between the photocurrent and applied X-ray dose rate under the reverse bias voltages of 60 V, 90 V, and 120 V, respectively, with figure insets showing the time-dependent device responses. In this experiment, the shutter of the X-ray source was alternately opened and closed for periods of 30 s with a fixed dose rate of 7 mGy/s. Similar to inorganic X-ray detectors, our devices showed linear relationships between the photocurrent and dose rate following the Fowler model [19].

It should be noted that the X-ray-induced photocurrent of the 0.010 wt.% SWNT device increased suddenly at -120 V (Figure 3c). Furthermore, the time-dependent response of this device showed a non-saturating behavior, and it was enhanced as reverse bias voltage increased. This unexpected behavior of the device can be explained through band diagram analysis. Figure 3d represents the band diagram of the fabricated X-ray detector structure. The work function of the top (LiF/Al) and bottom (Au) electrodes is 4.2 and 5.1 eV [20, 21], respectively. The lowest unoccupied molecular orbital and highest occupied molecular orbital levels of SY are 2.8 and 5.0 eV, respectively [22, 23]. When the pure p-type polymer is used as the active layer of the X-ray detector, the Schottky barrier heights are 2.3 eV for electrons and 0.8 eV for holes. Adding SWNTs (which has a work function of 4.5 eV) [20] in the active layer reduced the Schottky barrier heights for electrons and holes to 0.6 and 0.3 eV, respectively (Figure 3d). This changed the contacts from Schottky to near-Ohmic, which supply space-charge-limited current (SCLC) [24]. According to a previous report, the generation of SCLC influences the effective barrier height of a device and causes the formation of slower and non-saturating transients in the device [25]. We confirmed that such phenomenon was even more pronounced according to the increment of SWNT concentration.

**Figure 4:**
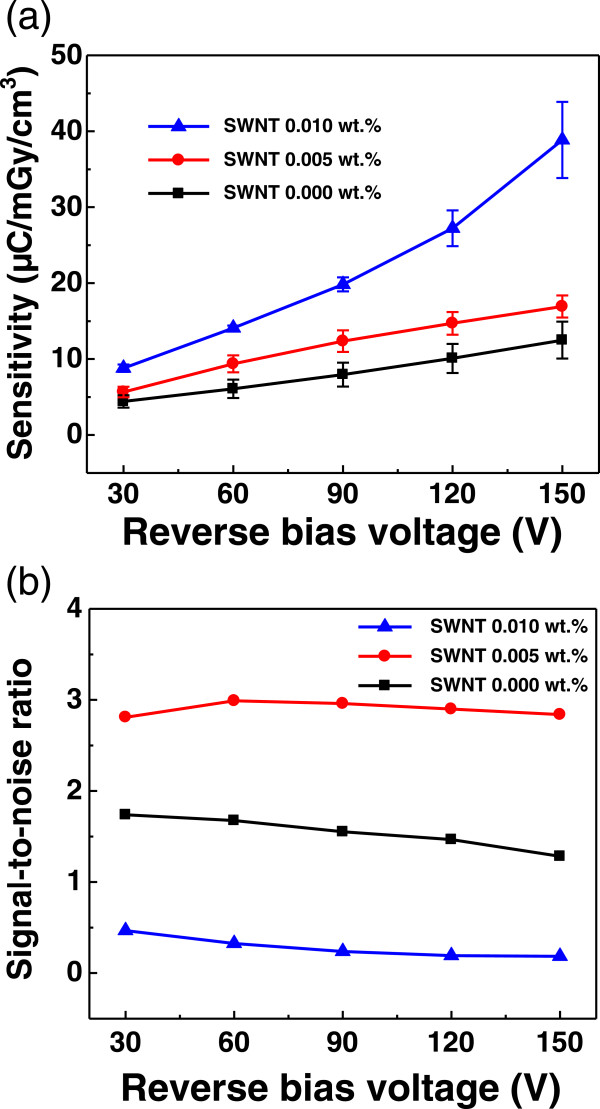
**X-ray detection sensitivity and signal-to-noise ratio of the fabricated devices. (a)** X-ray detection sensitivity and **(b)** signal-to-noise ratio of the devices with three different SWNT concentrations as a function of the reverse bias voltage.

X-ray detection sensitivity is an important factor for the evaluation of the X-ray detector. Figure 4a shows the calculated X-ray detection sensitivity as a function of reverse bias voltage for the devices with three different SWNT concentrations. The sensitivity of the device was obtained by dividing the slope of the photocurrent versus dose rate graph (Figure 3a,b,c) by the active volume of the device (3 mm × 3 mm × 5 μm). All of the devices showed positive correlation between the sensitivity and applied voltage, which was due to the longer carrier drift length of the X-ray-induced charges at high electric field strength [25]. Similar to the photocurrent, it was observed that the sensitivity of the devices increased when SWNTs were included in the active layer. At a reverse bias voltage of 150 V, for instance, the sensitivities of the devices with SWNT concentrations of 0, 0.005, and 0.01 wt.% were 12.5, 16.9, and 38.9 μC/mGy/cm^3^, respectively. This implies that our SWNT enriched polymer composite efficiently enhances the performance of the polymer-based X-ray detector.

For the realization of a practical X-ray detector, it is also required for the device to have a sufficiently large output photocurrent compared to the dark current. Therefore, we calculated the SNR of our device, which was defined as the ratio between the photocurrent and dark current. Figure 4b shows the SNR as a function of reverse bias voltage for the devices with three different SWNT concentrations under a fixed X-ray dose rate of 7 mGy/s. Generally, the SNR should be greater than 1, which means that the generated photocurrent should be greater than the dark current. From this point of view, the 0.010 wt.% SWNT device showed the lowest SNR value (approximately 0.18 at -150 V) although it had the highest sensitivity. As shown in Figure 3, at high SWNT concentration, the electrical contacts between the electrodes and active layer were changed from Schottky to near-Ohmic. This change led to the increase of leakage current under dark conditions which resulted in a low SNR value at high SWNT concentration. Therefore, an appropriate concentration of SWNT should be selected to enhance the sensitivity and guarantee a minimum SNR at the same time. Our experimental result indicated that 0.005 wt.% is the optimal SWNT concentration rather than 0.01 wt.% because it shows enhanced performance in both sensitivity and SNR compared to that of the device without SWNTs.

**Figure 5:**
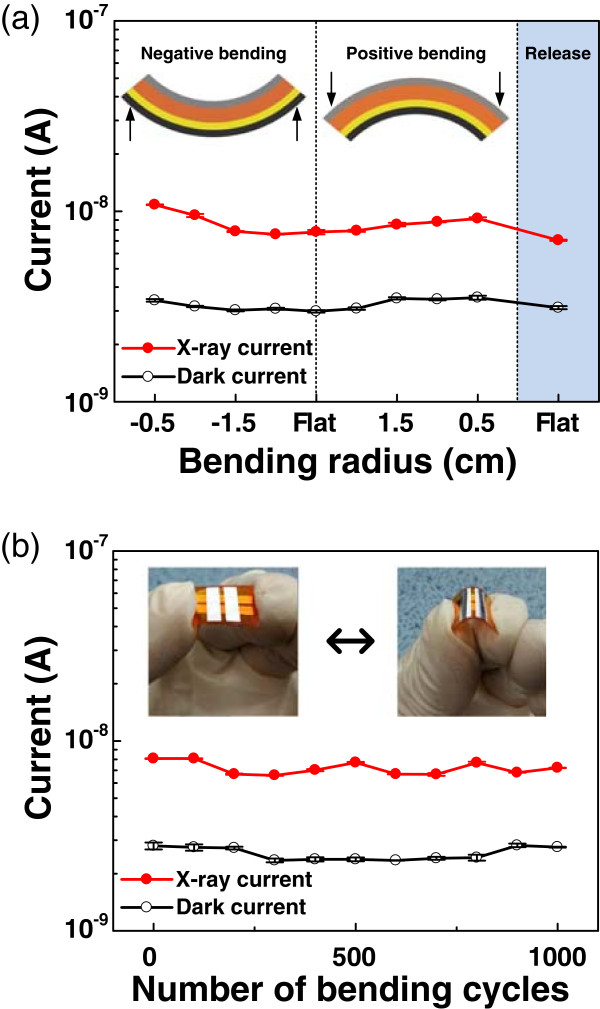
**Device stability tests under various bending conditions. (a)** Dark current and X-ray-induced current of the 0.005 wt.% SWNT device as a function of bending radius. The dark current remained similar despite the bending radius, but the X-ray-induced current changed slightly. **(b)** Dark current and X-ray-induced current of the 0.005 wt.% SWNT device after repeated bending cycles. The device showed a stable response for up to 1,000 bending cycles.

Figure 5a shows dark currents and X-ray-induced currents of the 0.005 wt.% SWNT device under bending conditions with various radii. In this measurement, the X-ray dose rate was 7 mGy/s and the reverse bias voltage was 120 V. It should be noted that the dark currents exhibited no discriminable response to changes in the bending radius, even though the X-ray-induced currents were slightly changed. The different responses may be attributed to the changes in surface morphology and the effective intensity of X-ray irradiation on the device [26]. Figure 5b shows the bending stability of the device in the same condition as that of Figure 4a. The device was bent 1,000 times, with a bending radius of 0.5 cm in the positive direction, and both dark current and X-ray-induced current were measured after every 100 cycles of bending. These results confirm that the device exhibited a stable X-ray response with good mechanical flexibility.

## Conclusions

In summary, we fabricated a polymer-based flexible X-ray detector and enhanced the performance using a SWNT enriched polymer composite as an active layer. The X-ray-induced photocurrent and the sensitivity were enhanced as the SWNT concentration of the composite layer increased. However, at high SWNT concentration, the speed and stability of the response decrease due to the reduction in the Schottky barrier height. The optimum SWNT concentration was determined in consideration of both sensitivity and SNR. With the SWNT enriched polymer composite active layer, we demonstrated the mechanical flexibility of the device which shows a stable X-ray response. We expect that our device will be used in medical imaging and non-destructive analysis as a next-generation flexible X-ray detector.

## Electronic supplementary material

Additional file 1: **Supporting information.** A PDF document containing figures showing optical images and I-V characteristics of the composite film. (PDF 129 KB)
