# Supplementary material for: Enhancement of X-ray detection by single-walled carbon nanotube enriched flexible polymer composite
Source: Nanoscale Res Lett. 2014 Nov 12;9:610. doi: 10.1186/1556-276X-9-610 (PMC4493992; doi:10.1186/1556-276X-9-610)
Supplement: Supplementary file 1 — Additional file 1: Supporting information. A PDF document containing figures showing optical images and I-V characteristics of the composite film. (PDF 129 KB) [file 11671_2014_2482_MOESM1_ESM.pdf]

Supporting Information for Publication

Enhancement of X-ray Detection by Single-Wall Carbon Nanotubes Enriched Flexible Polymer Composite

Heetak Han,[1] Sanggeun Lee,[1] Jungmok Seo,[1] Chandreswar Mahata,[1] Sung Hwan Cho,[2] A-Reum Han,[3] Keun-Sung Hong,[3] Joon-Ho Park,[3] Myung-Jin Soh,[3] Cheolmin Park,[2] and Taeyoon Lee[1*]

(1) Nanobio Device Laboratory, School of Electrical and Electronic Engineering, Yonsei University, 50 Yonsei-ro, Seodaemun-Gu, Seoul 120-749, Republic of Korea

(2) Nano-Polymers Laboratory, Department of Materials Science and Engineering, Yonsei University, 50 Yonsei-ro, Seodaemun-Gu, Seoul 120-749, Republic of Korea

(3) Luxen Technologies, Inc., 396 Worldcupbuk-ro, Mapo-Gu, Seoul 121-795, Republic of Korea

* Corresponding author:

Tel: +82-2-2123-5767

Fax: +82-2-313-2879

e-mail address: taeyoon.lee@yonsei.ac.kr

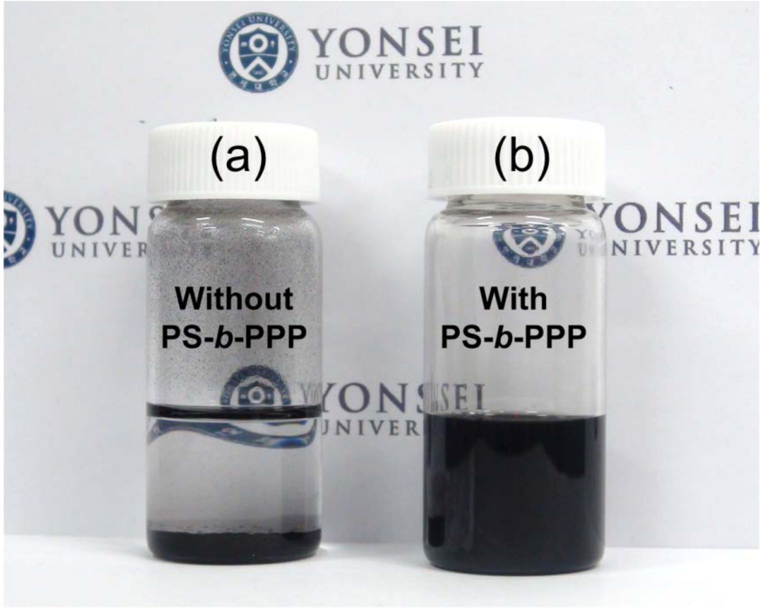

Figure S1 Photograph of 0.1 mg/mL of SWNT dispersion toluene solutions. SWNT dispersion solutions (a) without and (b) with PS-*b*-PPP dispersant

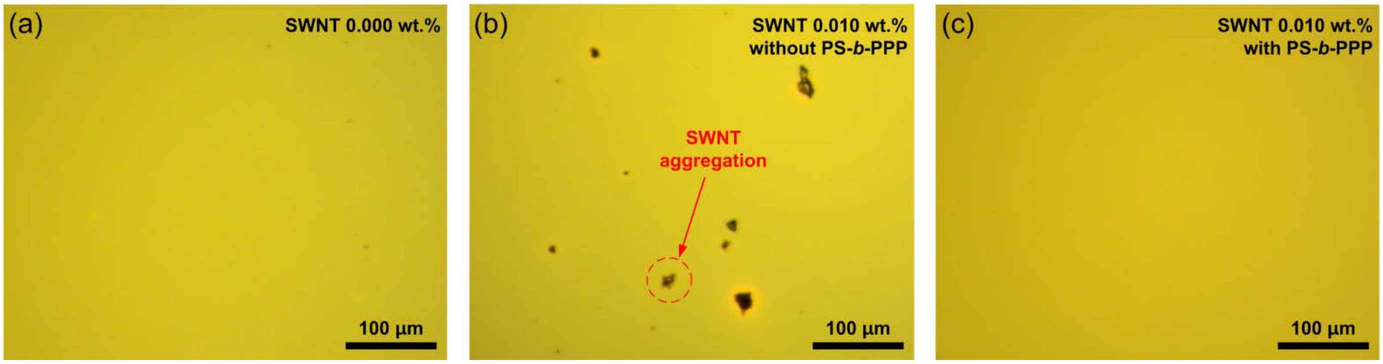

Figure S2 Optical microscope images of SWNTs enriched polymer composite films. The composite films (a) without SWNT, (b) with 0.010 wt.% SWNT, and (c) with 0.010 wt.% SWNT and PS-b-PPP dispersant. The SWNTs formed large aggregates and bundles in the composite films without dispersant. In contrast, there were no significant aggregations of SWNTs on the composite films with PS-b-PPP dispersant.

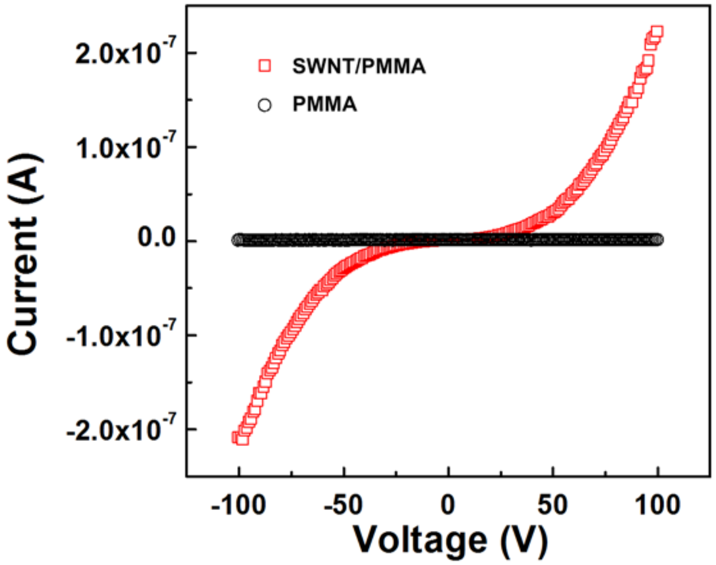

Figure S3 *I*-*V* characteristics of PMMA and SWNT/PMMA devices. SWNT/PMMA device (SWNT 0.1 wt.%) showed ambipolar characteristics.
